# Whole genome sequence of the syntrophic fatty acid-degrading bacterium *Syntrophomonas curvata* GB8-1^T^

**DOI:** 10.1128/mra.01525-25

**Published:** 2026-02-13

**Authors:** Ethan Humm, Neil Q. Wofford, Jessica R. Sieber, Marco Morselli, Matteo Pellegrini, Michael J. McInerney, Sung Min Ha, Robert P. Gunsalus

**Affiliations:** 1 Department of Microbiology, Immunology and Molecular Genetics, University of California 5116, Los Angeles, California, USA; 2 Department of Microbiology and Plant Biology, University of Oklahoma 6187, Norman, Oklahoma, USA; 3 Department of Molecular, Cell and Developmental Biology, University of California 5116, Los Angeles, California, USA; 4 UCLA DOE Institute, University of California 5116, Los Angeles, California, USA; 5 Department of Integrative Biology and Physiology, University of California 5116, Los Angeles, California, USA; DOE Joint Genome Institute, Berkeley, California, USA

**Keywords:** *Syntrophomonas*, genomes, syntrophs, anaerobes

## Abstract

The syntrophic fatty acid-degrading bacterium *Syntrophomonas curvata* strain GB8-1**^T^**, isolated from anaerobic granular sludge, metabolizes C4–C18 fatty acids when grown with a suitable hydrogenotrophic partner. The complete genome sequence data provide insight into syntrophic fatty acid compound degradation under thermodynamically limiting conditions when exogenous electron acceptors are absent.

## ANNOUNCEMENT

Members of the genus *Syntrophomonas* within the family Syntrophomonadaceae participate in anaerobic biomass recycling in natural and anthropogenic anaerobic habitats limited for exogenous electron acceptors (1, 2). The syntrophic microbial process is not well understood at the genetic or biochemical level. *Syntrophomonas curvata* GB8-1**^T^** (=DSM 15682^T^) was isolated from an up-flow anaerobic sludge blanket (UASB) reactor treating beer wastewater in Beijing, China, using crotonate as the sole source of carbon and energy (3, 4). This gram-negative bacterium grows on straight-chain fatty acids (C4–C18) when co-cultured with a suitable hydrogenotrophic partner such as *Methanobacterium formicicum* DSM 1535^T^ (3).

*S. curvata* GB8-1**^T^** was obtained from the DSMZ and stocked in the McInerney laboratory culture collection. It was cultivated at 37°C in a mineral-salts medium (DSMZ medium 213) containing 20 mM crotonate till late-log phase (3, 4). DNA was extracted using the Zymo D4081 Quick-DNA Magbead Plus Kit per the manufacturer’s instructions. DNA was fragmented using g-Tubes (Covaris, cat# 520079) in an Eppendorf 5424 centrifuge for 60 s at 3,000 × *g* for a total of 3 spins according to the manufacturer’s protocol. The average size of the sheared DNA was then calculated using the genomic DNA assay (TapeStation-Agilent Technologies) for the construction of genomic libraries. Sheared DNA was then concentrated using AMPure PB beads according to the manufacturer’s recommendations (PacBio-Multiplexed microbial libraries using SMRTbell Express Template Prep Kit 2.0, #101-696-100 version, 07 July 2020). Library preparation was performed according to the PacBio protocol. DNA sequencing was performed using the PacBio Sequel IIe platform, which generated 56,620 raw reads. Demultiplexing and adapter trimming were done using Lima v2.9.0 (https://github.com/pacificbiosciences/barcoding). All reads were then used for assembly by Canu v2.2 (5). Assembled genomes were further refined by Circlator v1.5.5 (6) to identify circular contigs, remove redundant non-circular contigs, and rotate circular contigs to start with *dnaA*. A completeness check was performed by CheckM v1.0.18 (7), and the N50 quality was determined by Assembly stats v1.01 (https://github.com/sanger-pathogens/assembly-stats). Genome ORF calling and annotation were performed by NCBI PGAP v6.10 (8). All tools were run with default parameters.

Properties of the *S. curvata* GB8-1**^T^** genome are shown in Table 1, which comprises one circular contig with a total length of 3,228,016 bp with three rRNA operons.

**TABLE 1:** *Syntrophomonas curvata* GB8-1 ^**T**^ genome information

Feature	Genome value
Topology	Circular
Size (bp)	3,228,016
GC%	47.70%
Protein coding genes	2,921
16S number	3
tRNA number	48
Coverage	35.97×
Raw reads	56,620
High-quality reads	8,730
N50 quality value (bp)	3,228,016
Completeness value	99.83%
16S similarity^*a*^	99.55–99.59%
Isolation source	UASB reactor treating beer wastewater
Location	Tsinghua University, Beijing, China
NCBI taxonomy ID	231025
DSM collection	15682^T^

^*a*^ Genomic 16S similarity to the published 16S accession no. NR_025752.1 determined by NCBI BLASTN 2.17.0+ (9). The range represents differences among the three 16S genomic sequences.

The genome of *S. curvata* GB8-1**^T^** contains multiple paralogs of each of the beta-oxidation enzymes to accomplish fatty acid activation and metabolism to acetate, suggesting the ability to degrade a range of aliphatic organic acid compounds (1, 2). It also possesses multiple gene clusters for hydrogenase and formate dehydrogenase enzymes to dispose of protons and electrons as hydrogen or formate. Also present is a gene for membrane-bound electron transfer flavoprotein-methylmenaquinone oxidoreductase (EMO) needed to catalyze thermodynamically difficult reverse electron transfer reactions involving acyl-CoA oxidation (10, 11).

## Data Availability

The complete genome sequence of *Syntrophomonas curvata* GB8-1^T^ has been deposited in GenBank under assembly accession GCA_052976825.1, and the raw sequencing reads have been deposited under the accession no. SRR35930697. The IMG genome OID number is 8025903884, and the IMG submission ID is 303512.
